# Evolution of C-point singularities and polarization coverage of Poincaré–Bessel beam in self-healing process

**DOI:** 10.1038/s41598-024-67582-w

**Published:** 2024-07-18

**Authors:** Subith Kumar, Anupam Pal, Arash Shiri, G. K. Samanta, Greg Gbur

**Affiliations:** 1 Physical Research Laboratory, Ahmedabad, Gujarat 380009 India; 2 Indian Institute of Technology Gandhinagar, Ahmedabad, Gujarat 382424 India; 3 Department of Physics and Optical Science, University of North Carolina Charlotte, Charlotte, NC 28223 USA

**Keywords:** Optics and photonics, Optical physics, Optical techniques

## Abstract

As a vector version of scalar Bessel beams, Poincaré–Bessel beams (PBBs) have attracted a great deal of attention due to their non-diffracting and self-healing properties as well as the presence of polarization singularities. Previous studies of PBBs have focused on cases that consist of a superposition of Bessel beams in orthogonal circular polarization states; here, we present a theoretical and experimental study of PBBs for which the polarization states are taken to be linear, which we call a linear PBB. Using a mode transformation of a full Poincaré beam constructed from linear polarization states, we observe the linear PBB as providing an in-principle infinite number of covers of the Poincaré sphere in the transverse plane and with an infinite number of C-points with positive and negative topological indices. We also study the dynamics of C-point singularities in a linear PBB in the process of self-healing after being obstructed by an obstacle, providing insight into “Hilbert Hotel” style evolution of singularities in light beams. The present study can be useful for imaging in the presence of depolarizing surroundings, studying turbulent atmospheric channels, and exploring the rich mathematical concepts of transfinite numbers.

## Introduction

Beams with nontrivial tailoring of their spatial profile, now called spatially structured optical beams or structured light, have attracted a great deal of attention recently due to their wide range of applications. These include optical manipulation^1,2^, micromachining^3–5^, imaging^6,7^, and optical communications^8,9^. Typically, structured beams are generated through mode conversion of a Gaussian beam and can include modulation of the amplitude, phase, and polarization of the beam. A beam with a uniform state of polarization is referred to as a scalar beam, and a beam with a nonuniform state of polarization is referred to as a vector beam, or sometimes a Poincaré beam.

Perhaps the most familiar class of structured beams are scalar optical vortex beams, which possess an optical vortex on their central axis. An optical vortex is a line of zero intensity around which the phase has a circulating or helical structure; the study of these and related singularities now form a field known as singular optics^10^. In a transverse plane, an optical vortex manifests as a point, and the phase always changes by an integer multiple of 2*π* around the vortex; this multiple is called the topological charge. The topological charge is generally a conserved quantity and is typically only created or annihilated in pairs of equal and opposite charges. Vector vortex beams may be generated through the superposition of vortex beams of different orders in orthogonal polarization states. The typical singularities of vector beams are C-points, points of circular polarization in a transverse plane at which the orientation of the polarization ellipse is undefined. In analogy to topological charge, polarization singularities always possess a topological index that takes on half-integer values, and C-points have an index of ± 1*/*2.

A *full* Poincaré beam is a special type of vector vortex beam, ideally containing all polarization states that are present on the surface of the Poincaré sphere. Such beams have found practical application; for example, they have lower scintillation than comparable beams of uniform polarization in the presence of atmospheric turbulence^11,12^. Efforts have been made to find a general method to estimate the polarization coverage of Poincaré beams and the parameters influencing the polarization coverage^13^ in order to broaden the scope of such beams for different applications.

Bessel beams^14–16^ are a different class of structured light beams that have found application in various areas of optics^17–20^, owing to their high intensity extended focus, finite beam width, nondiffractive propagation over considerable distances, and self-healing properties downstream of obstacles. Bessel beams of non-zero order also possess an optical vortex on their central axis, making them another class of vortex beams.

Recently, there has been much interest in generalizing scalar Bessel beams into vector Bessel beams^21,22^ for use in studying quantum effects^23,24^, microscopy and imaging^25,26^, and propagation in turbulent media^11,12,27^. Research has been done on imparting the ideally 100% polarization coverage of full Poincaré beams to Bessel beams through the generation of Poincaré- Bessel beams, and the structure and propagation characteristics of such beams have been studied^28,29^, including self-healing^30^. However, Poincaré–Bessel beams are typically generated by the superposition of a pair of Bessel beams with orthogonal circular polarization states, which results in a single C-point singularity on the beam axis. When linear orthogonal polarization states are used instead, the resultant beams possess infinite sets of positive and negative charge C-points in their cross-section, and their topological and polarization structures are significantly different. Efforts have been made^31^ to demonstrate light fields with infinite topological charge using the solution of the paraxial Helmholtz equation involving a Gaussian function and an arbitrary analytical function. However, these beams lack structural stability during propagation.

In this paper, we report a theoretical and experimental study of the characteristics of a linear Poincaré–Bessel beam. Using an axicon, we have transformed a Poincaré beam in a rectangular polarization basis, with polarization coverage > 98%, into a Poincaré–Bessel beam. The study of the polarization characteristics of this Poincaré–Bessel beam reveals a number of noteworthy features. These features include each ring of the Poincaré–Bessel beam behaving like a Poincaré beam with polarization coverage > 75%, self-healing of the polarization structure, and a degree of polarization that is independent of the beam obstruction. Furthermore, using a map of the polarization ellipse orientation, we study the dynamics of the infinite set of C-point singularities in the self-healing process and consider its connections to the mathematics of infinite sets.

## Poincaré–Bessel beams

We begin by discussing the theoretical characteristics of Poincaré–Bessel beams relevant to our investigations, including their polarization singularities and their polarization coverage on the Poincaré sphere. Monochromatic non-diffracting Bessel beams were first introduced in 1987 by Durnin as the precise solution of the scalar wave equation^32^ In cylindrical coordinates, the complex amplitude of the beam contains a *l*th order Bessel function, and the beam is known as a Bessel beam of order *l*, which at any propagation distance *z* has the form,

1 $$U_{l} \left( {r,\varphi ,z} \right) = {\text{exp}}\left[ {ik_{z} z} \right]J_{l} \left( {k_{r} r} \right){\text{exp}}[il\varphi ],$$

where *k*_*r*_*, k*_*z*_ are the transverse and longitudinal components of the wavevector *k* = 2*π/λ*, respectively, with *k*^2^ = *k*_*r*_^2^ + *k*_*z*_^2^. The variables *φ* and *r* represent the azimuthal angle and the radial distance from the axis in the cross-section of the beam, respectively, and *J*_*l*_ is the *l*th order Bessel function of the first kind. Here, we have assumed the conventional exp[− *iωt*] dependence. Such beams are non-diffracting because their angular spectrum representation consists entirely of plane waves with the same longitudinal wavevector component *k*_*z*_. An ideal Bessel beam has an infinite amount of energy in its cross-section, so in practice, Bessel beams are generated with a Gaussian envelope; this results in them being non-diffracting only over a finite propagation distance.

Poincaré–Bessel beams may be represented as a vector superposition of coherent Bessel modes with orthogonal polarizations. Typically, these orthogonal states are taken to be circular polarization states. In our experiment, we have used the superposition of 0th and 1st order Bessel beams with perpendicular linear polarizations in the form,

2 $${\mathbf{E}}_{0} \left( {\mathbf{r}} \right) = {\text{exp}}\left[ {ik_{z} z} \right][iJ_{0} \left( {k_{r} r} \right){\hat{\mathbf{x}}} + J_{{1}} \left( {k_{r} r} \right)e^{ \pm i\varphi } {\hat{\mathbf{y}}}].$$

The extra phase factor of *i* in the first term rotates the polarization pattern of the field by 90°, placing the resulting singularities on the horizontal axis for convenience.

Both constituent Bessel beams have the same longitudinal wavenumber *k*_*z*_, and the beam as a whole is, therefore, propagation invariant. Because of the mixture of different space and polarization degrees of freedom, the state of polarization varies throughout the cross-section of the beam, and beams of this type are generally referred to as vector beams.

In a transverse electromagnetic field, the polarization is defined by the state of the electric field vector **E**(**r**), which can be characterized by its complex Cartesian components *E*_*x*_ and *E*_*y*_. Determining the polarization state of the field **E**(**r**) is equivalent to finding the geometric properties of its corresponding ellipse.

The geometric properties of the polarization ellipse are not directly measurable. In 1852, however, Stokes introduced four experimentally measurable parameters, now known as the Stokes parameters, which can be used to relate the field and geometric properties of polarization^33^,

3 $$\begin{gathered} S_{0} = |E_{x} |^{2} + |E_{y} |^{2}, \hfill \\ S_{{1}} = |E_{x} |^{2} - |E_{y} |^{2}, \hfill \\ S_{{2}} = {\text{2Re}}\{ E_{x} Ey ^* \} , \hfill \\ S_{{3}} = - {\text{2Im}}\{ E_{x} Ey ^* \} . \hfill \\ \end{gathered}$$

Spanning a 3-D space by the unit Stokes parameters (*S*_1_*, S*_2_*, S*_3_) results in a unit sphere known as the Poincaré sphere, which provides a complete geometrical illustration of the polarization state of light (see Chapter 7 of^10^). Any point on the surface of the Poincaré sphere represents a distinct, fully polarized state. North and South poles correspond to the left- and right-handed circular polarization, respectively, and all points on the equator of the sphere represent linear polarization states. Vector beams with spatially inhomogeneous distribution of polarization vector in a transverse plane will have states that cover some portion of the Poincaré sphere and are often also called Poincaré beams. In the special case in which the entire surface of the Poincaré sphere is represented in a transverse plane, the beam is referred to as a full Poincaré beam^34^. In the cross-section of a paraxial vector beam, two types of singularities typically arise. C-points are points of circular polarization where the orientation of the polarization ellipse is undefined; L-lines are lines of linear polarization where the handedness of the polarization ellipse is undefined. C-points are topologically robust singularities that are most often considered for applications, so we focus on them in this paper.

The location of C-points in a vector field can be readily determined by writing Eq. (2) in the circular polarization basis, with

4 $$\hat{e}_{ \pm } = \frac{{\hat{x} \pm i\hat{y}}}{\surd 2}$$

leading from Eq. (2) to the expression

5 $$\begin{aligned} {\varvec{E}}_{0} \left( {\varvec{r}} \right) & = \frac{i}{\sqrt 2 }exp\left[ {ik_{z} z} \right]\left\{ {\left[ {J_{0} \left( {k_{r} r} \right) - J_{1} \left( {k_{r} r} \right)e^{i\varphi } } \right]\hat{e}_{ + } } \right. \\ & \quad \left. { + \left[ {J_{0} \left( {k_{r} r} \right) + J_{1} \left( {k_{r} r} \right)e^{i\varphi } } \right]\hat{e}_{ - } } \right\}, \\ \end{aligned}$$

C-point singularities will appear at points where the field is circularly polarized, i.e., at points where either of the orthogonal components in Eq. (5) vanish. If the amplitude of the ${\widehat{e}}_{+}$ component vanishes, the field will be right-circularly polarized, and if the amplitude of the ${\widehat{e}}_{-}$ component vanishes, the field will be left-circularly polarized.

On inspection of Eq. (5), we can see that these components can only vanish at points on the *x*-axis, or *φ* = 0*, π*. This leads to the expression

6 $$\begin{aligned} {\varvec{E}}_{0} \left( {{\varvec{r}},\left\{ {0,{\varvec{\pi}}} \right\}} \right) & = \frac{i}{\sqrt 2 }exp\left[ {ik_{z} z} \right]\left\{ {\left[ {J_{0} \left( {k_{r} r} \right) \mp J_{1} \left( {k_{r} r} \right)e^{i\varphi } } \right]\hat{e}_{ + } } \right. \\ & \quad \left. { + \left[ {J_{0} \left( {k_{r} r} \right) \pm J_{1} \left( {k_{r} r} \right)e^{i\varphi } } \right]\hat{e}_{ - } } \right\}, \\ \end{aligned}$$

where the upper sign represents *φ* = 0 and the lower sign *φ* = *π*. The zeros, therefore lie on the *x*-axis at points where

7 $$J_{0} \left( {k_{r} r} \right) + J_{{1}} \left( {k_{r} r} \right) = 0,\quad J_{0} \left( {k_{r} r} \right) - J_{{1}} \left( {k_{r} r} \right) = 0.$$

There are an infinite number of solutions of these two equations with respect to *r*, and the solutions alternate between them as *r* increases. The solution with the lowest *r* satisfies the equation with the minus sign. Linear PBBs are the first beams we have encountered that have an infinite and stable number of singularities in their cross-section. Infinite singularities have been encountered before, in the generation of fractional vortex beams by a fractional spiral phase plate^13,35^, but this infinite set of singularities only arises for an exact half-integer fractional order of the phase plate. In a linear PBB, in contrast, the singularities are stable under beam perturbations: if one of the constituent Bessel beams has its amplitude slightly changed, for example, this will result in a slight change of singularity position but all the singularities will remain. A linear PBB is therefore an ideal beam for investigating relationships between the mathematics of infinite sets and optical singularities.

Let us label the solutions of Eq. (7) by an integer *n*, with positive *n* representing *φ* = 0 and negative *n* representing *φ* = *π* (*n* = 0 is unused). Then the *n*th zero satisfies

8 $$J_{0} \left( {k_{r} r_{n} } \right) + \left( { - {1}} \right)^{n} J_{{1}} \left( {k_{r} r_{n} } \right) = 0.$$

Using Eq. (3) for *S*_3_, and substituting from Eq. (5), we readily find that in general we have

9 $$S_{{3}} \left( {\mathbf{r}} \right) = - {\text{2cos}}\varphi J_{{1}} \left( {k_{r} r} \right)J_{0} \left( {k_{r} r} \right).$$

Now we let *φ* = 0*, π* and look at the value of *S*_3_ for each zero *r*_*n*_. Using Eq. (7), we find that

10 $$S_{{3}} \left( {r_{n} } \right) = \pm {2}|J_{0} \left( {k_{r} r_{n} } \right)|{2}\left( { - {1}} \right)^{n} .$$

This indicates that the polarization singularities on the *x*-axis alternate in handedness, with the + *x*-axis starting with a right-handed C-point and the -*x*-axis starting with a left-handed C-point.

However, it can also be shown (and will be seen explicitly in the simulations that follow) that the topology of all C-points on the + *x*-axis are stars, whereas the topology of all C-points on the -*x*-axis are lemons. The net topological index of the Poincaré–Bessel beam is effectively the sum of plus infinity and minus infinity and is formally undefined. This naturally raises questions about the topological behavior of Poincaré–Bessel beams, which we explore in the next section.

It is to be noted that we will never in practice see an infinite number of singularities in a beam’s cross-section, as the beam intensity decreases as we move away from the beam axis, and singularities will eventually be undetectable in the low intensity outskirts. We will qualify our statements by referring throughout the paper to an “in-principle infinite” number of singularities.

## Simulations of Poincaré–Bessel beam self-healing

In recent years, it has been shown that fractional vortex beams exhibit behavior analogous to transfinite mathematics as the fractional vortex charge of the beam is changed^36^. In particular, such beams evolve an infinite number of vortex pairs of opposite sign, and then each member of a pair annihilates with its opposite neighbor, leaving an extra unbalanced charge in its wake, in a process comparable to the classic example of Hilbert’s Hotel. This behavior has been demonstrated experimentally^13,37^. It can be argued that a fractional vortex beam violates topological charge conservation and creates a new unbalanced charge by passing through a state with an infinite number of positive and negative charges, making the topological charge of the beam undefined. As a Poincaré–Bessel beam is a vector beam with an infinite number of singularities of positive and negative indices, it is natural to ask whether a similar Hilbert Hotel can be produced in such beams. This question was a significant motivation for the research of this paper.

Because it is comprised of scalar Bessel beams, a Poincaré–Bessel beam will exhibit self-healing after being partially obstructed by an obstacle, reconstructing its original form after propagating a sufficient distance. This process must also reconstruct any polarization singularities, but if an odd number of singularities are blocked at the beam’s center, the net blocked topological index alone does not characterize the total number of singularities in the blocked region, and it is of interest to see how these singularities reappear in the healing process.

To investigate the evolution of polarization singularities during self-healing, we consider the propagation of the Poincaré- Bessel beam expressed in Eq. (2) obstructed by an off-axis circle defined as

11 $$circ\left( {{\varvec{r}} - {\varvec{r}}_{0} } \right) = \left\{ {\begin{array}{*{20}c} {1,\quad\left| {r - r_{0} } \right| > a} \\ {0,\quad\left| {r - r_{0} } \right| \le a } \\ \end{array} } \right.$$

where *a* is the radius of the circle, **r** is the position vector in the transverse plane, and **r**_0_ is the radial position of the center of the block. The Poincaré–Bessel beam was given the transverse wavevector *k*_*r*_ = 3*.*83*/l*, where *l* = 76*.*5 µm. The choice of *a* and **r**_0_ are taken to obstruct an odd number of singularities near the beam center.

**Figure 1:**
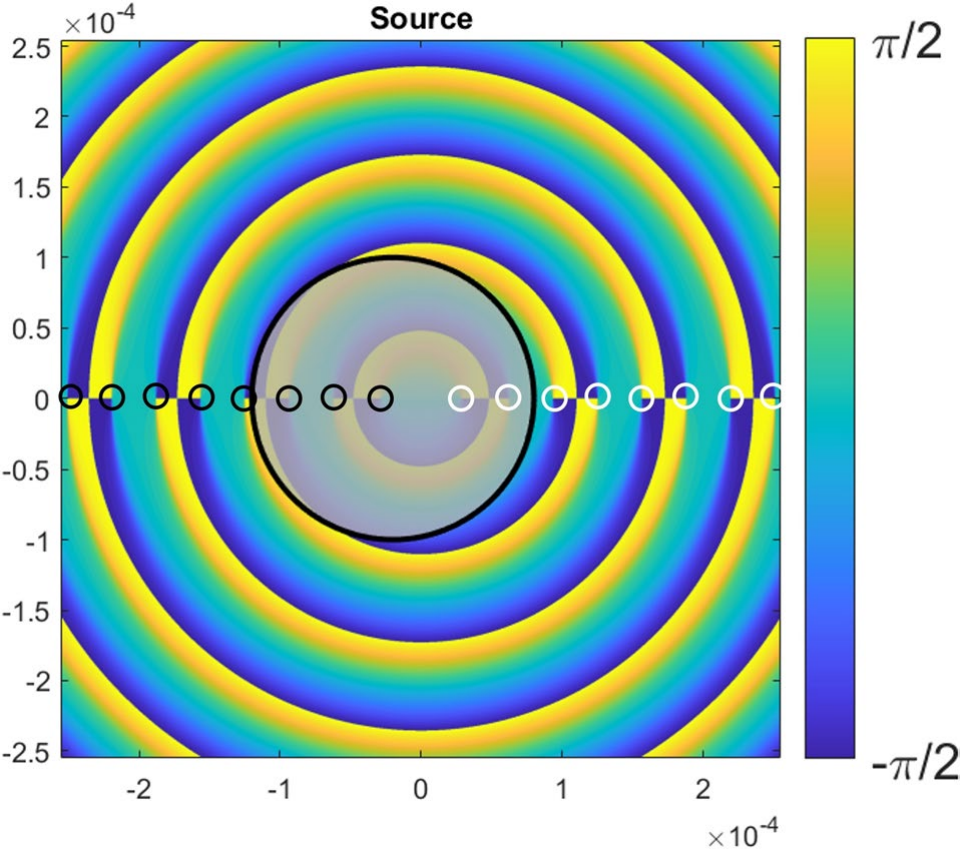
Stokes vector phase of the obstructed Poincaré–Bessel beam of Eq. (2). The opaque circle shows the extent of the region covered by the spherical obstruction. The lemon and star singularities are marked by black and white circles, respectively.

Figure 1 shows the Stokes vector phase (orientation angle) before the block. Singularities are points where all the phase values converge to a point. Singularities where the angle increases/decreases in a clockwise path around them are lemons/stars, respectively. The values of *a* = 0*.*1 mm and **r**_0_ = (0*.*02 mm*,* 0) are chosen such that an odd number of singularities are blocked by the obstacle (see the black shaded region of Fig. 1), three lemons and two stars with a net topological index of *n* = + 1*/*2.

We may evaluate the propagation of the blocked beam using Fresnel propagation, integrating over the shifted position vector **R** = **r** − **r**_0_ in the source plane,

12 $${\varvec{E}}\left( {\user2{r^{\prime}}, z} \right) = \frac{{e^{{ik_{z} z}} }}{i\lambda z}\smallint \smallint circ\left( {\varvec{R}} \right){\varvec{E}}_{0} ({\varvec{R}} + {\varvec{r}}_{0} ) \times exp\left[ {\frac{ik}{{2z}}\left| {\user2{r^{\prime}} - \left( {{\varvec{R}} + {\varvec{r}}_{0} } \right)} \right|^{2} } \right]d^{2} R,$$

where *λ* is the wavelength of the beam and **r** and **r′** are the position vectors on the source and detector planes, respectively. This integral cannot be evaluated analytically, and even computational analysis is a challenge, as a Bessel beam is a beam of theoretically infinite width, yet any simulation must use a finite integration window. With a sufficiently large window, however, we found that numerical integration yields results that agree well with the experiment.

**Figure 2:**
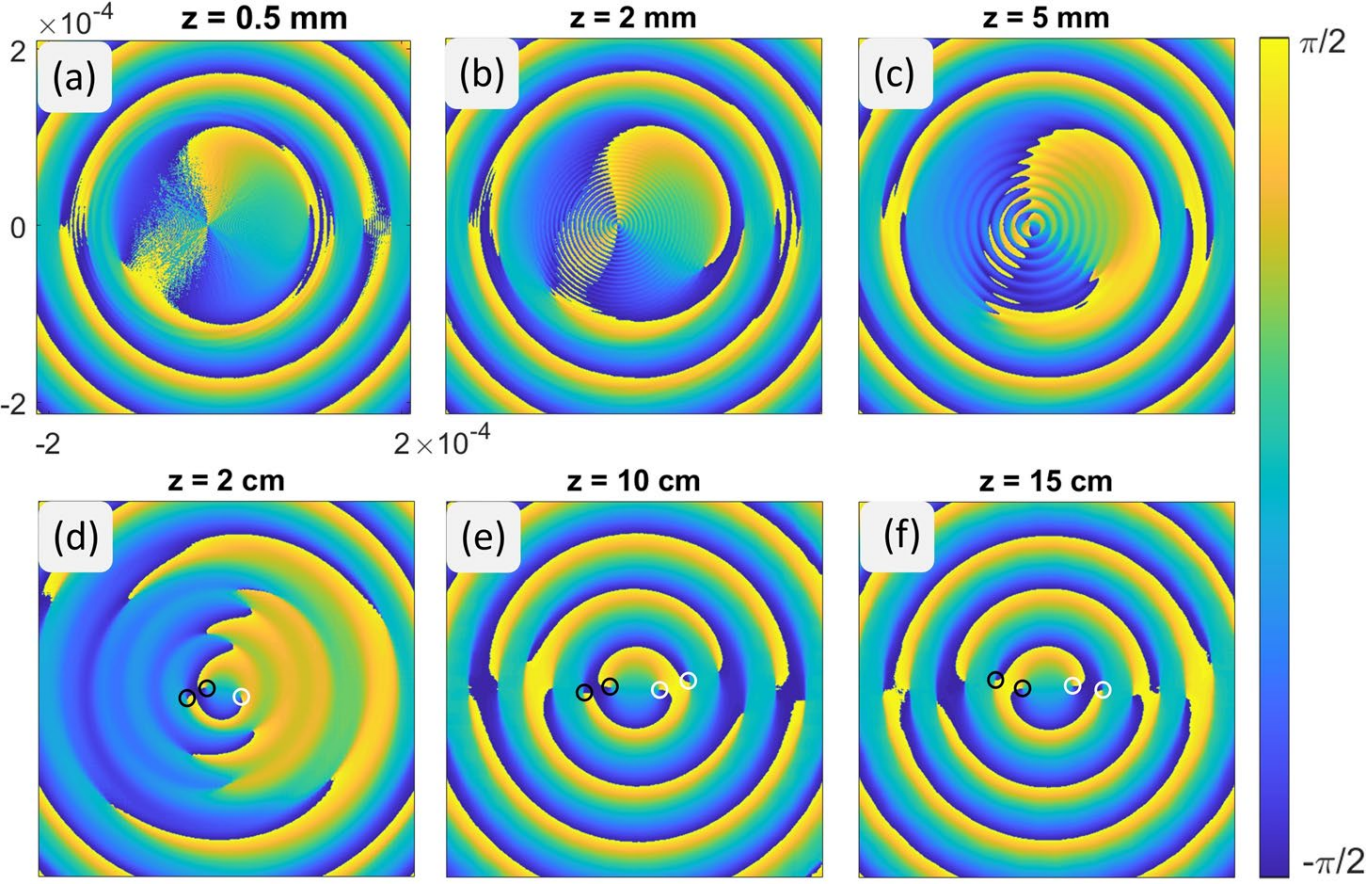
Hilbert Hotel-like evolution of polarization singularities in the self-healing process of the Poincaré–Bessel beam after passing an obstruction.

Figure 2 shows the Stokes vector phase (orientation angle) of the beam at different propagation distances from 0*.*5 mm to 15 cm after the block. As was shown in Fig. 1, three lemon and two star singularities were blocked by the obstruction. The net topological index *n* = +1*/*2 is conserved in this process, as the orientation angle of the Stokes vector phase around the outside of the block region still increases by *π*, but the precise number and types of singularities within the obstructed region are lost. These singularities must reappear during the self-healing of the Bessel beams, and it is of interest to see topologically how this happens.

Figure 2a shows that the beam possesses a central topological index of *n* = +1*/*2 immediately after the block. In Fig. 2b,c, however, we see that the beam has manifested a high frequency circular “ripple” that contains many lemon-star singularity pairs. These pairs annihilate as the ripples spread out, the result of which can be seen in Fig. 2c, and by 10 cm of propagation distance in Fig. 2d the original pre-obstruction pattern of singularities has returned, with only minor distortions remaining. This equity of positive and negative singularities is then preserved in propagation, as evident from Fig. 2f.

Computationally, it appears that there is a localized Hilbert Hotel type mechanism occurring in the finite region of the block; such a mechanism has previously only been seen in fractional vortex beams^36^. Immediately after the block, the field is zero in the block shadow and may be interpreted as a non-generic singularity of polarization extended over an area in space. This zero area then breaks into an infinitely dense region of singularity pairs that gradually annihilate until only singularities associated with the original beam remain. This evolution is analogous to Hilbert’s original discussion of a hotel of infinite rooms, in which guests (negative index) leave rooms (positive index) and move to the next room over, leaving a single extra room at the end.

This mechanism is a new demonstration of infinite mathematics arising in an optical problem and distinct from the fractional vortex beams mentioned earlier, where the infinite singularities are distributed in the infinite cross-section of the beam. Here, we have an infinite number of singularities densely packed in a finite beam region.

Because we used a computational method to evaluate the propagation of the Poincaré–Bessel beam after the block, we cannot rigorously quantify the number of singularities and their evolution on propagation. An analytic approach for studying the beam singularities immediately after the block is underway and will be presented in future work.

## Experimental details

**Figure 3:**
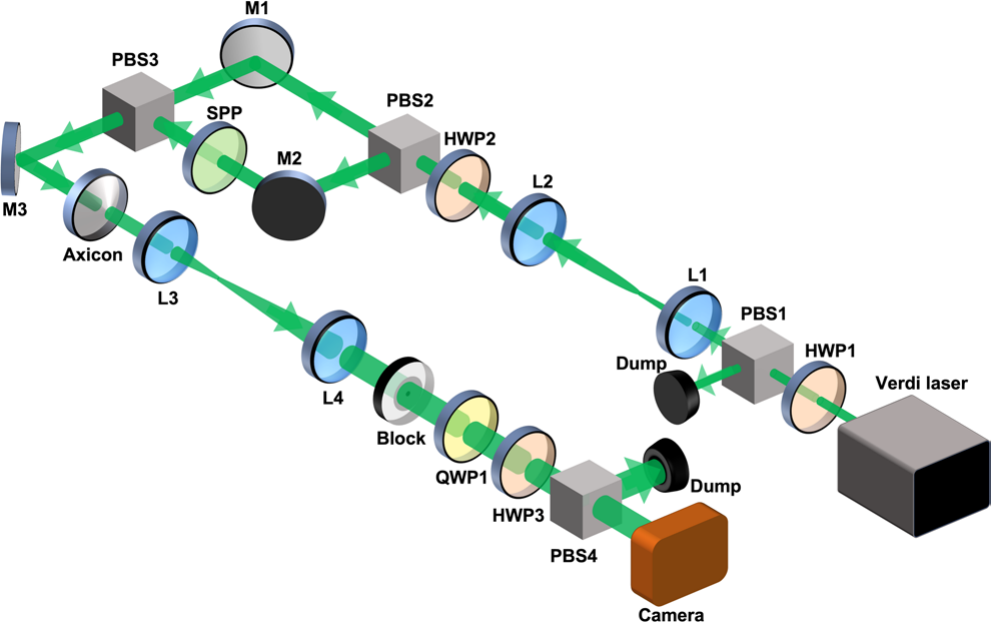
Experimental setup for the generation of Poincaré–Bessel beam. HWP1-3: *λ/*2 plates; PBS1-4: polarizing beam splitter cubes; SPP: spiral phase plate; QWP1: *λ/*4 plate; M1-3: mirrors; L1-4: lenses.

The schematic experimental scheme for the generation and study of a linear Poincaré–Bessel beam is shown in Fig. 3. A continuous wave (cw), single-frequency, green laser (Coherent, Verdi V10) providing maximum output power of 10 W in *TEM*_00_ spatial profile with *M*^2^ < 1*.*1 at 532 nm is used as the primary laser source. Operating the laser at its maximum output power for reliable system performance, we have used a power attenuator comprised of the combination of a *λ/*2 (HWP1) and a polarizing beam splitter (PBS1) cube to control the laser power in the experiment. A pair of plano-convex lenses, L1 and L2, of focal length, *f*_1_ = 50 mm and *f*_2_ = 200 mm, respectively, placed in 2 *f*_1_–2 *f*_2_ configuration are used to expand the laser beam. The *λ/*2 (HWP2) plate is used to control the relative intensity between the two arms of the Mach–Zehnder interferometer (MZI) configured with two plane mirrors, M1 and M2, and two PBSs, PBS2 and PBS3. A full Poincaré beam is generated^34^ by placing a spiral phase plate (SPP) in one of the arms (here between mirror, M2, and PBS3) of MZI. The SPP has a transverse thickness variation corresponding to the phase variation of the vortex order of *l* = 1. The superposition of the vertically polarized Gaussian beam from the reflected arm of the MZI, on propagation through the SPP, and the horizontally polarized Gaussian beam of the transmitted arm on the PBS3 produces the full Poincaré beam with electric field,

13 $$E_{0} \left( {\mathbf{r}} \right) = \alpha U_{G}^{0} \left( {r,\phi } \right){\hat{\mathbf{x}}} + \beta U_{LG}^{l} \left( {r,\phi } \right){\hat{\mathbf{y}}}$$

where *U*_*G*_^0^ is a Gaussian beam and U_LG_^*l*^ is a Laguerre–Gauss beam of radial order 0 and azimuthal order *l*, *r* and *φ* are the radial and azimuthal coordinates, and *α* and *β* are the relative amplitudes of the orthogonal polarization modes of the FP beam, satisfying *α*^2^ + *β*^2^ = 1. The values of *α* and *β*, can be controlled by varying the angle, *θ*, of the HWP2 angle by *α* = cos *θ/*2 and *β* = sin *θ/*2.

This full Poincaré beam then propagates through the Axicon with an apex angle of 196*°*, transforming it into a Poincaré- Bessel beam. The Poincaré–Bessel beam is then expanded with the second pair of plano-convex lenses, L3 and L4, of focal lengths of *f*_3_ = 50 mm and *f*_4_ = 300 mm, respectively in 2 *f*_3_–2 *f*_4_ imaging configuration. The polarization state of the beam is characterized using the standard Stokes measurement technique^38^ with the help of a quarter-wave plate (QWP), HWP3, PBS4, and the CCD camera. For the self-healing study, an obstacle (Block) made of a microscope cover slip with a black dot of diameter 0.2 mm at the center is used in the experiment.

## Results and discussion

### Characteristics of linear Poincaré–Bessel beam

First, we studied the polarization characteristics of the linear Poincaré–Bessel beam generated through the mode transformation of the full Poincaré beam by the axicon. On propagation through the axicon, the input full Poincaré beam, represented by Eq. (13), was transformed into a Poincaré–Bessel beam; the orders and polarizations of the Bessel beams match those of the components of the input full Poincaré beam. Therefore, the electric field of the Poincaré–Bessel beam can be represented as

14 $${\mathbf{E}}_{0} \left( {\mathbf{r}} \right) = {\text{U}}_{0} [\alpha J_{0} \left( {k_{r} r} \right) + \beta J_{{1}} \left( {k_{r} r} \right)e^{il\varphi } ].$$

The maximum electric field amplitude at the origin is represented by the constant *U*_0_. The radial wave vector *k*_*r*_ can be written in terms of the wave vector *k,* axicon apex angle *γ,* and refractive index *n* as *k*_*r*_ = *k*(*n − *1) cos(*γ*).

**Figure 4:**
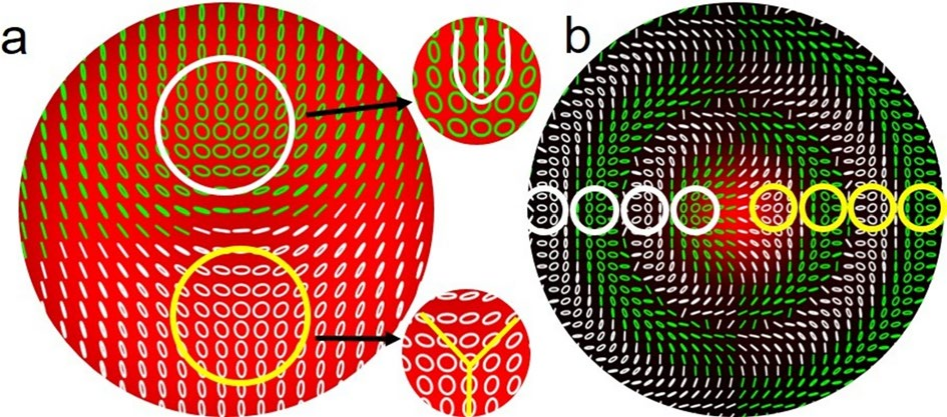
Polarization distributions of (**a**) full Poincaré and (**b**) Poincaré–Bessel beams calculated using the experimental parameters in Eqs. 13 and 14. The insets are the magnified images of lemon and star polarization singularities marked by white and yellow circles.

Using the experimental parameters (diameter (FWHM) of Gaussian and vortex beams as ~ 3.4 mm and ~ 6.8 mm, respectively, and their relative intensity *α/β* = 1) in Eqs. (13) and (14) and the theoretically calculated Stokes parameters, we have calculated the orientation *ψ* and ellipticity *χ*^38^ of the polarization ellipse of the input full Poincaré beam and corresponding Poincaré–Bessel beams. The results are shown in Fig. 4. As evident from Fig. 4a, the transverse polarization distribution of the input beam of vortex order *l* = 1 contains C-point polarization singularities in the form of a lemon (see the white circle) and star (see the yellow circle) pair and a single L-line, confirming the vortex order of the full Poincaré beam to be *l* = 1, the same as seen in our recent report^39^.

To make this identification clear, the insets of Fig. 4 show a zoomed-in detail of the polarization singularities. Throughout the rest of the manuscript, we identify the white and yellow circles as the lemon and star polarization singularities, respectively.

**Figure 5:**
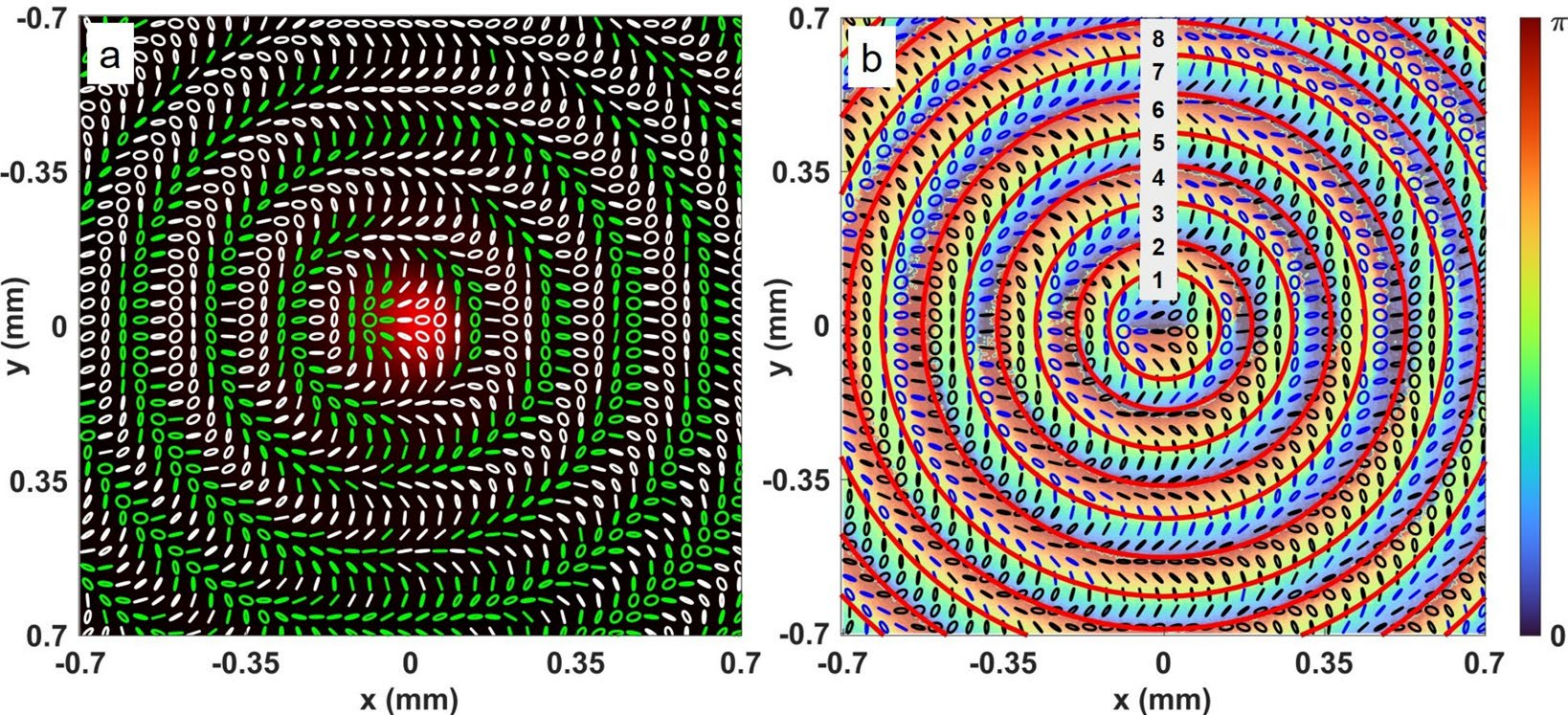
Experimentally measured polarization distribution of Poincaré–Bessel beam with (**a**) beam intensity and (**b**) ellipse orientation map at the background. The red colour rings identify the characteristic rings of the Poincaré–Bessel beams. The rings are also marked integer numbers for further studies.

However, the polarization distribution of the Poincaré–Bessel beam, as shown in Fig. 4b, shows lines of polarization singularities, where pairs of lemon (white circle) and star (yellow circle) singularities lie on concentric circles. As the Bessel beam has a characteristic intensity distribution of concentric rings and, in-principle, infinite spatial extent, the Poincaré–Bessel beam, in-principle, carries infinite pairs of lemon and star singularities. A careful observation of the polarization distribution indicates that each of the rings of the Poincaré–Bessel beam contains a large number of polarization states, and the same polarization states repeat in all the rings, suggesting that the Poincaré–Bessel beam consists of an infinite number of full Poincaré beams. To confirm these interesting polarization characteristics experimentally for the Poincaré–Bessel beam, we recorded the intensity distribution for different polarization projections (using the combination of *λ*/4 and *λ*/2 plates at different combinations of the angles, the PBS). Using these intensity distributions, we have calculated the Stokes parameters, *S*_1_, *S*_2_, and *S*_3_, and measured the orientation *ψ* and ellipticity *χ* of the polarization ellipse. The results are shown in Fig. 5. As evident from Fig. 5a, the generated Poincaré–Bessel beam has a polarization distribution in close agreement with the theoretical results (see Fig. 4b). As expected, the Poincaré–Bessel beam generated by the full Poincaré beam has a central intensity maxima, and each ring contains a large number of polarization states.

To gain further perspective, we have recorded the polarization ellipse orientation (0 − *π*) of the Poincaré–Bessel beam along with the polarization distribution. The results are shown in Fig. 5b. As evident from Fig. 5b, the ellipse orientation pattern of each ring (marked by red colour and identified by the numbers 1, 2, 3…) has two singular points with opposite polarization ellipse orientation (counterclockwise, star and clockwise, lemon directions) confirming the presence of pair of polarization singularities in each ring and an infinite number of polarization singularity pairs in the transverse spatial distribution of the Poincaré–Bessel beam.

**Figure 6:**
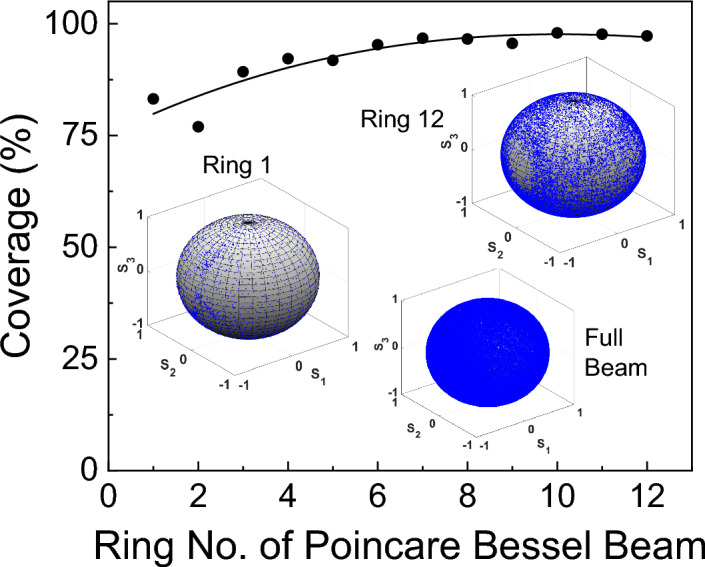
The variation of polarization coverage of each ring of Poincaré–Bessel beam. Inset images show the polarization distribution of ring 1, ring 12, and the entire Poincaré–Bessel beam on the Poincaré sphere.

Recently, we have devised a new method to estimate the polarization coverage of the full Poincaré beam^39^. Using the same treatment here, we have calculated the polarization coverage of each ring of the Poincaré–Bessel beam as marked in Fig. 5b. The results are shown in Fig. 6. As evident from Fig. 6, the polarization coverage of the first ring of the Poincaré–Bessel beam is > 75%. However, there is an increase in polarization coverage with ring number, and finally, all rings have polarization coverage > 97%. Although we expect to have the same polarization coverage in all rings, relatively lower polarization coverage for the central ring of the Bessel beam arises as an experimental artifact: the small number of useful camera pixels in the measurement process arising from the smaller spatial extent of the central ring. The slightly lower polarization coverage for the second ring is due to the asymmetry in the central lobe of the first-order Bessel beam generated from the vortex. The artifacts gradually die out with the increase in camera pixel numbers in accordance with the increase in the spatial extent of the Bessel rings. Therefore, we observe the initial increase of polarization coverage, which finally saturates, resulting in the same value for all rings, as expected.

For further support this, we have calculated the area (number of pixels) varying from 4*.*8 × 10^*−*2^ mm^2^ (2019) to 53*.*7 × 10^*−*2^ mm^2^ (23,633) for Ring number 1 to Ring no. 12 of the Poincaré–Bessel beam. However, as the coverage of more than 75% of the Poincaré sphere is deemed acceptable as a full Poincaré beam for many applications^40^, we can safely say that each ring of the Poincaré–Bessel beam is a full Poincaré beam. We have also presented the polarization Poincaré sphere of each ring of Poincaré–Bessel beam as the inset of Fig. 6. As expected, the Poincaré sphere, as evident from the inset of Fig. 6, gets populated with the number of points for the Bessel beam rings away from the center without increasing the polarization coverage substantially.

Finally, the polarization coverage of the entire linear Poincaré–Bessel beam, as also seen from the inset of Fig. 6, is around 100% and contains a large number of data points on the corresponding Poincaré sphere. Since the polarization states present in each ring of the Poincaré–Bessel beam covers the entire surface of the Poincaré sphere, one can imagine the polarization coverage of the whole Poincaré–Bessel beam as the superposition of an infinite number of full Poincaré spheres, resulting in a net polarization coverage the same as the single ring. This striking property of linear Poincaré–Bessel beams supports the self-healing characteristics of polarization coverage, the same as the intensity self-healing of the Bessel beam.

### Self-healing of Poincaré–Bessel beam

With the polarization properties of the beam well understood, we further studied the intensity and polarization self-healing properties of the Poincaré–Bessel beam. For the intensity self-healing study, we have recorded the intensity profile of the Bessel beam generated through the Gaussian, scalar vortex of order, *l* = 1, and the full Poincaré beams as input to the axicon. On the other hand, we have estimated the degree of polarization, ellipse orientation, and ellipticity of the Poincaré–Bessel beam using the Stokes parameters calculated from the intensity profile of the Bessel beam recorded for different polarization projections. As observed previously^29,30^, here we also observe that the zero-order Bessel beam, first-order Bessel beams, and Poincaré–Bessel beam start regaining and maintaining their initial spatial intensity and polarization distribution after a propagation distance of *d* = 10 cm with complete healing at *d* = 59 cm. It is interesting to note that the beam obstruction, although disturbs the polarization distribution of the beam, has negligible or no impact on the degree of polarization of the beam. The Poincaré–Bessel beam maintains a high degree of polarization (≈ 1) throughout its cross-section.

**Figure 7:**
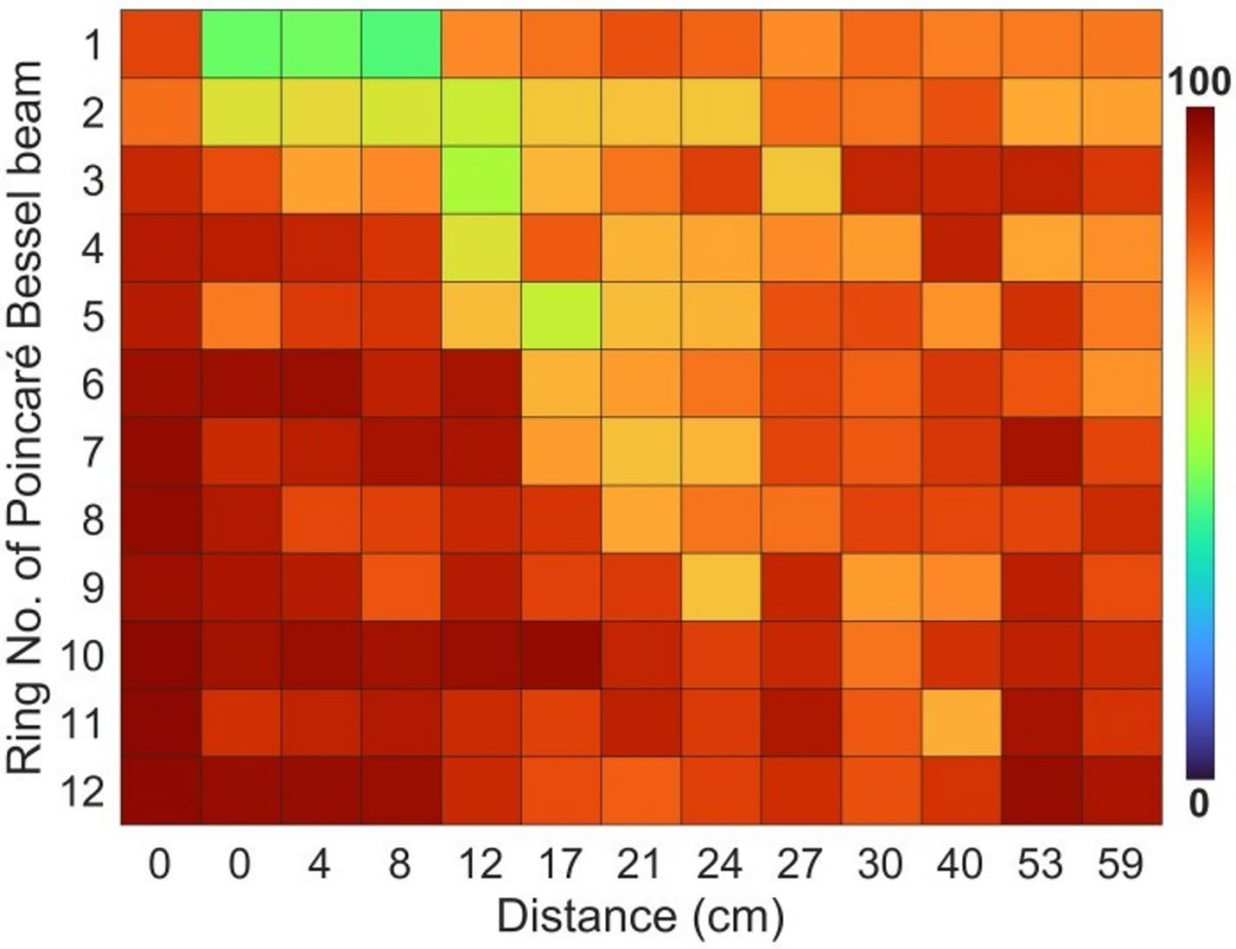
Variation of polarization coverage of different rings of the Poincaré–Bessel beam along propagation during the self-healing process. The lower polarization coverage due to the beam obstruction is gradually moving in the outward direction during the self-healing process.

To understand further, we have estimated the polarization coverage of each ring along the propagation distance. The results are shown by the colour chart in Fig. 7. The rows and columns of Fig. 7 represent the ring number and propagation distance, respectively. It is evident from Fig. 7 that all the rings before the beam obstruction carry polarization coverage > 84%, as already seen in Fig. 6. After the obstruction, the polarization coverage of the central ring is low due to the obstruction, while the polarization coverage of the outer rings remains unaffected. However, the disturbance in the polarization coverage of the beam gradually travels away from the inner ring to the outer rings as the beam propagates, and finally, the beam regains high polarization coverage for all rings. This observation indicates that the self-healing of the polarization coverage of the Poincaré–Bessel beam occurs due to the energy flow of the Bessel beam from the outward rings to the inside rings.

We also blocked the beam at various positions on the transverse plane and calculated the degree of polarization and polarization distribution to understand the impact of beam block sites on the self-healing process. We have selected obstructions of two sizes (circles of diameter 0.7 mm and 0.5 mm) at two different positions. In both cases, it is observed that although the degree of polarization is unperturbed by the beam obstruction, obstruction mediated polarization disturbance gradually spreads away from the disturbed site before returning to the initial polarization distribution.

### Polarization singularities

**Figure 8:**
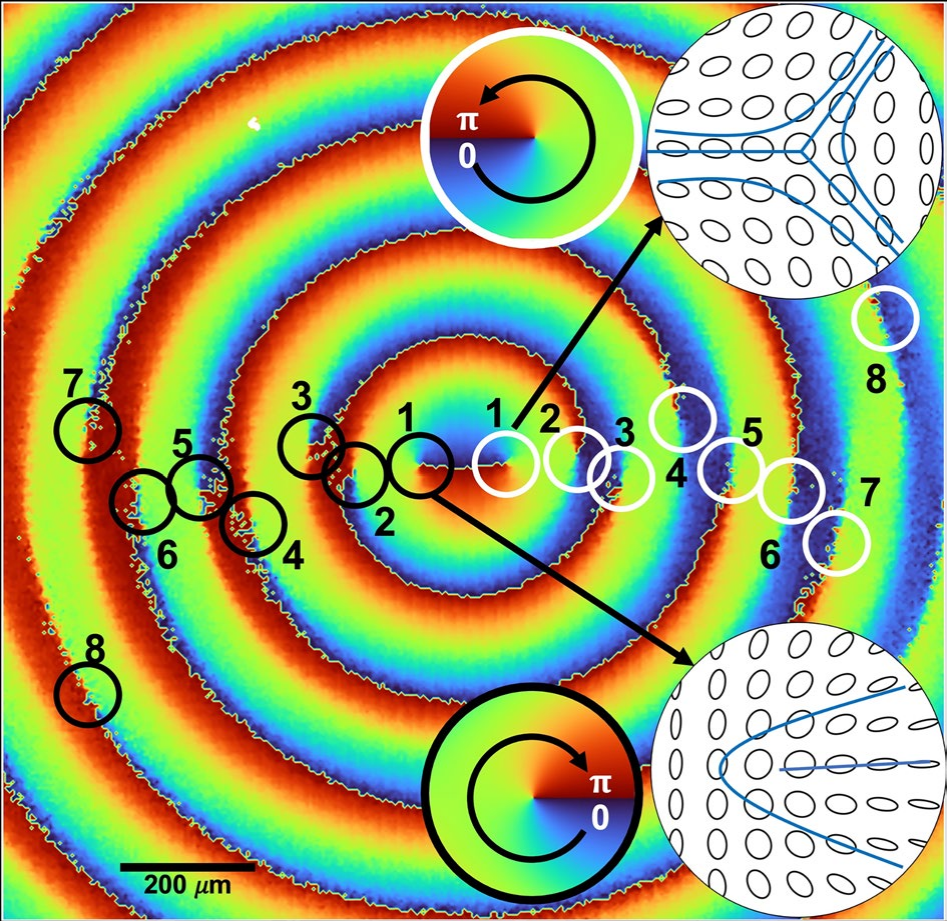
Polarization ellipse orientation map of the Poincaré–Bessel beam showing the infinite series of C-point singularity pairs. The white and black circles identify the star (polarization ellipse varying from 0 − *π* in the counterclockwise direction) and lemon (polarization ellipse varying from 0 − *π* in the clockwise direction) singularities, respectively. The insets show the polarization distribution and corresponding polarization ellipse orientation at C-point singularities.

With the polarization and intensity properties of the Poincaré–Bessel beam characterized, we then studied the evolution of the C-point singularities of the beam in the self-healing process. As presented in Fig. 5a, the Poincaré–Bessel beam carries an in-principle infinite number of pairs of lemon and star singularities in close agreement with the theory (see Fig. 1). To identify the C-point singularities, we have calculated the orientation of the polarization ellipse varying from 0 - *π* with the results shown in Fig. 8. For clarity, we have identified the direction of ellipse orientation about the singularity point by 0 − *π* in the counterclockwise direction with white circles representing star singularities and the clockwise direction with black circles representing lemon singularities.

As predicted by our theoretical analysis, we observe the polarization ellipse orientation map of the Poincaré–Bessel beam in the rectangular basis to contain a lemon and star singularity pair in each ring. As ideal Bessel beams have infinite spatial extent and thus an infinite number of rings, we note that one can get an in-principle infinite sequence of lemon and star polarization singularity pairs by transforming the full Poincaré beam into a Poincaré–Bessel beam. In our experiment, with *l* = 1, we have one infinite sequence of lemon and star singularities. For more general Poincaré–Bessel beams, the number of sequences is decided by the number of polarization singularity pairs of the input full Poincaré beam, which is equal to the order of the vortex beam component. Experimentally, by using higher-order full Poincaré beams of vortex orders, *l* = 2, 3, and 4, we have observed the resultant Poincaré–Bessel beam to carry 2, 3 and 4 infinite sequences of lemon and star polarization singularity pairs, all oriented roughly along lines from the origin to the beam outskirts.

**Figure 9:**
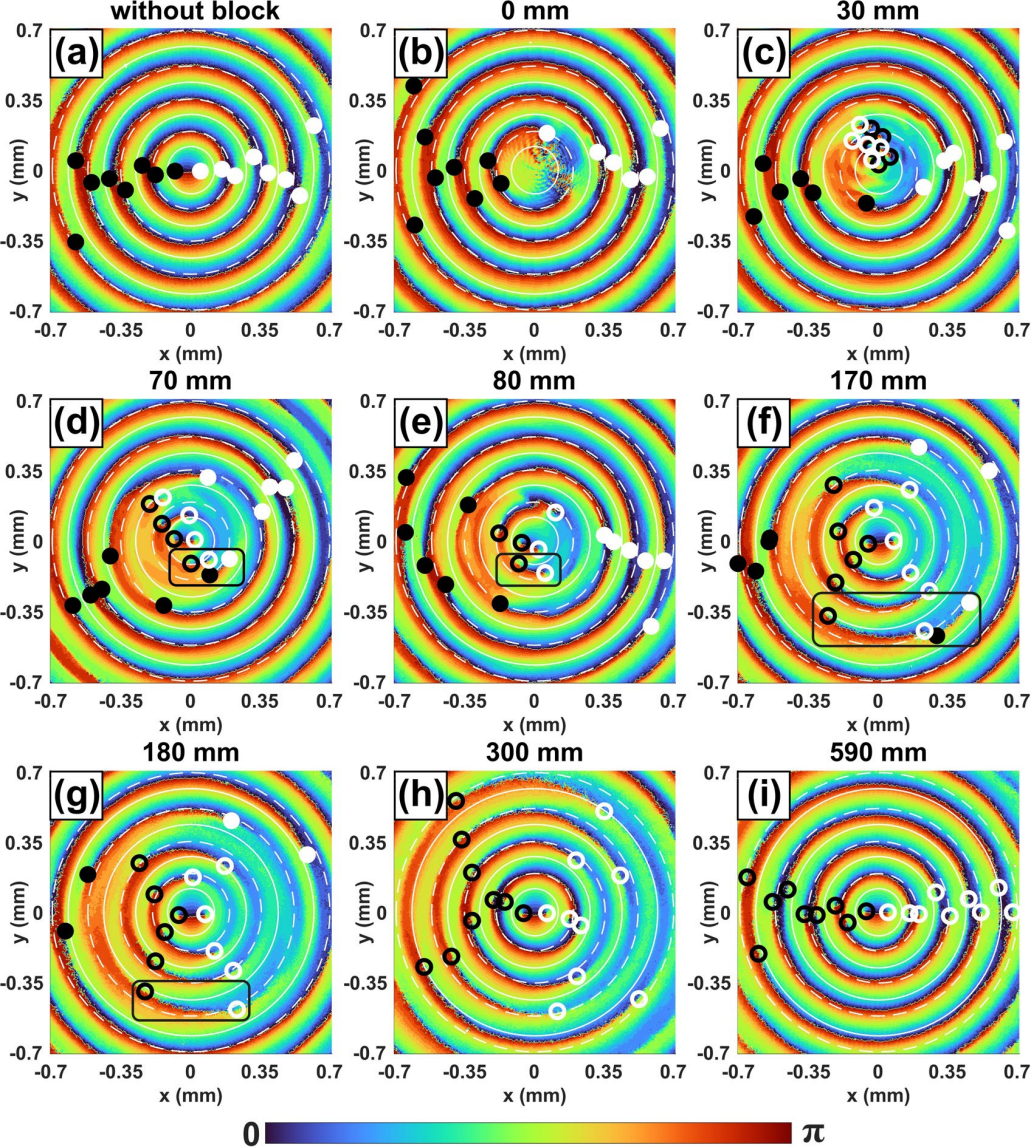
Observation of dynamics of the infinite number of C-point singularity pairs of the Poincaré–Bessel beam along propagation after the beam obstruction. The star and lemon C-point singularities of the initial beam are marked by white and black dots, respectively. The newly formed star and lemon singularity pairs due to beam obstruction are identified by white and black circles. The black rectangles mark the annihilation of the old singularity with the new singularity.

We have also measured the dynamics of the C-point singularities of the Poincaré–Bessel beam in the self-healing process. Using the intensity distribution of the beam for different projections along beam propagation, we have derived the polarization ellipse orientation map with the results shown in Fig. 9. As expected, the Poincaré–Bessel beam contained an infinite sequence of star and lemon polarization singularities, identified by the black and white dots in Fig. 9a. Because the lemons and stars have opposite topological indices, and there are an infinite number of them, the net topological index of this beam is technically undefined. (Roughly speaking, one would say it is “positive infinity plus negative infinity,” which is undefined.)

In the self-healing study, we selected the block position so that it created an asymmetry in the total number of star and lemon singularities. As evident from Fig. 9b, the block has removed two stars and one lemon singularity from the polarization ellipse orientation map at the center, confirming the presence of asymmetry in the number of singularities right after the beam block. The beam block-induced perturbation results in the production of a large number of new C-point singularity pairs, lemons, and stars, marked by black and white open circles (see Fig. 9c).

In Fig. 9c–e, we can clearly see the production of a large number of singularity pairs within the block region, as also found in our simulations in Fig. 2. This is what we believe to be a Hilbert Hotel-type creation of an infinite number of singularity pairs in the immediate shadow of the block; these pairs then annihilate until only the singularities needed to reconstruct the original beam remain.

Due to the limited spatial resolution arising from the beam size and the pixel size of the CCD, we have only marked a limited number of new C-point singularity pairs and observed their propagation dynamics. The newly generated singularity pairs (black and white open circles) not only annihilate with each other but also with the original singularity pairs (black and white dots) as the beam propagates (see Fig. 9d–i, and eventually they take over the original singularity pairs in the center (Fig. 9i).

After further beam propagation, the self-healed Poincaré–Bessel beam, as shown by Fig. 9i, regains the C-point singularities of the original beam and reproduces the polarization ellipse orientation map (see Fig. 9a). A close look at the dynamics of the Poincaré–Bessel beam in the self-healing process, especially the polarization ellipse orientation map of Fig. 9a,b,i, we see a general replacement of the original singularities (solid white and black circles) of the beam with singularities produced at the interior of the beam and propagating outward (hollow white and black circles). By the end of the process, all singularities, at least within the finite measurement region of the experiment, have been replaced by singularities generated after the block. The process does not seem to precisely match the Hilbert Hotel dynamics observed in fractional beams^13,36,37,41^, but is very reminiscent of it. The self-healing dynamics of the experimentally measured Poincaré–Bessel beam closely match the theoretical results presented in Fig. 2.

The beam, therefore, appears to possess two intermixed evolutions associated with an infinite number of singularity pairs. In the block region, an infinite number of singularity pairs are created, which eventually reproduce the singularities that were “erased” by the block. Outside the block region, new singularity pairs propagate outward to replace the original singularities. The result of both of these mechanisms is a reconstruction of the original singularity pattern and beam.

## Conclusion

In this work, we have studied the polarization characteristics of the linear Poincaré–Bessel beam and found close agreement between the experimental and computational results. We studied both the polarization coverage of the beam as well as its topological characteristics, including its self-healing after a block.

The use of a Poincaré beam in a rectangular basis containing all polarization states covered by the surface of the Poincaré sphere produces a Poincaré–Bessel beam with each ring having polarization coverage greater than 75%. The polarization coverage of the Poincaré–Bessel beam is the same or slightly higher than the polarization coverage of any of the rings. Therefore, one can consider the Poincaré–Bessel beam as the superposition of an infinite number of full Poincaré rings.

We have also investigated the topological characteristics of linear Poincaré–Bessel beams. As predicted, we see that the beams possess an infinite number of C-point (lemon and star) singularity pairs arranged along lines through the beam axis, and we have found that the number of such pairs is dependent on the number of C-point singularity (lemon and star) pairs present in the input full Poincaré beam. As the number of C-point singularity pairs of the full Poincaré beam is equal to the vortex order of the constituent superposed orthogonal polarized beams, one can generate any number of in-principle infinite sequences of C-point singularity (lemon and star) pairs by simply adjusting the vortex order.

We have confirmed that the Poincaré–Bessel beams exhibit self-healing characteristics after obstruction by a finite obstacle. It is striking that there are two distinct topological processes that arise during the self-healing process: the creation of a seemingly infinite number of singularity pairs in the shadow of the block that rapidly annihilate and the replacement of the unobstructed singularities by new singularities created after obstruction. Poincaré–Bessel beams appear to be an ideal class of beams to study the relationships between topological singularities of wavefields and the mathematics of infinite sets, and these relationships will be explored further in future work.

The rich and robust topological structure of linear Poincaré–Bessel beams has the potential to be used for a number of applications, including the imaging of objects in the presence of depolarizing surroundings and the creation of robust turbulent atmospheric channels for communication.

## Data Availability

The data that support the findings of this study are available from the corresponding author upon reasonable request.
